# Comparing the direct normal form and multiple scales methods through frequency detuning

**DOI:** 10.1007/s11071-018-4534-1

**Published:** 2018-09-14

**Authors:** A. J. Elliott, A. Cammarano, S. A. Neild, T. L. Hill, D. J. Wagg

**Affiliations:** 1 School of Engineering, University of Glasgow, Glasgow, G12 8QQ UK; 2 Department of Mechanical Engineering, University of Bristol, Bristol, BS8 1TR UK; 3 Department of Mechanical Engineering, University of Sheffield, Sheffield, S1 3JD UK

**Keywords:** Nonlinear, Vibration, Normal form, Multiple scales

## Abstract

Approximate analytical methods, such as the multiple scales (MS) and direct normal form (DNF) techniques, have been used extensively for investigating nonlinear mechanical structures, due to their ability to offer insight into the system dynamics. A comparison of their accuracy has not previously been undertaken, so is addressed in this paper. This is achieved by computing the backbone curves of two systems: the single-degree-of-freedom Duffing oscillator and a non-symmetric, two-degree-of-freedom oscillator. The DNF method includes an inherent detuning, which can be physically interpreted as a series expansion about the natural frequencies of the underlying linear system and has previously been shown to increase its accuracy. In contrast, there is no such inbuilt detuning for MS, although one may be, and usually is, included. This paper investigates the use of the DNF detuning as the chosen detuning in the MS method as a way of equating the two techniques, demonstrating that the two can be made to give identical results up to $\varepsilon ^2$ order. For the examples considered here, the resulting predictions are more accurate than those provided by the standard MS technique. Wolfram Mathematica scripts implementing these methods have been provided to be used in conjunction with this paper to illustrate their practicality.

**Electronic supplementary material:**

The online version of this article (10.1007/s11071-018-4534-1) contains supplementary material, which is available to authorized users.

## Introduction

In recent years, there has been substantial interest in the study of backbone curves, due to their utility in studying lightly damped nonlinear vibrations in multi-degree-of-freedom (MDOF) mechanical structures. The motivation for this paper comes from observations made by the authors when comparing backbone curves found using the multiple scales (MS) method (see, for instance, [1]) and those found using the normal form method, defined in [2].

The normal form method in [2] was developed as a technique that can be applied *directly* to systems of weakly coupled second-order nonlinear differential equations. This concept is not entirely uncommon, having previously been proposed in [3], but it is the matrix formulation proposed in [2] that is considered particularly beneficial to the current work. We will call this the “direct” normal form (DNF) method^1^ in order to differentiate it from the “classical” method described, for example, by Jezequel and Lamarque [4], Arnold [5], Murdock [6], Kahn and Zarmi [7] and Nayfeh [8]; the latter is not investigated here, as similar comparisons have previously been made, for example, in [2].

In recent years, the DNF method (and other normal form methods similar to this) has been used extensively to capture the responses of nonlinear systems. This includes, but is not limited to, describing modal interactions and bifurcations in backbone curves [9–12], recognising out-of-unison resonance in a taut cable [13], reduced-order modelling [14], nonlinear system identification [15, 16], investigating aeroelastic systems under fluid flow [17, 18], exploring applicability conditions for nonlinear superposition [19], and quantifying the significance of nonlinear normal modes [20]. In contrast with the recent development of the DNF method, the MS method is well established in the literature, with thorough discussions regarding its development readily available, for example, in [21–26].

Perturbation methods require the repeated application of a number of steps, building up an increasingly accurate solution by addressing smaller terms in each repetition. In the practical application of these methods, the steps can require significant computational effort and produce increasingly complex expressions, which can, arguably, hide the mathematical insight gained from employing such a technique. In this paper, we consider the “accuracy” of these methods by assessing the result after one or two repetitions of their respective steps. It is generally recognised that these techniques converge to the correct solution with many repetitions, so can ultimately be considered as precise as each other.

A contributing factor in the accuracy of the DNF method, as shown in [27], is the frequency detuning which arises in its formulation. In physical terms, this can be interpreted as a series expansion around the natural frequency of the underlying linear system. This is not naturally present in the MS technique; however, several examples of alternative detunings, applied to MS technique, can be found in the literature [28–33]. The attempt that most closely resembles the detuning of the DNF method is found in [32], although this proposed detuning is only employed in a small number of papers, such as [34, 35]. In [32], an $\varepsilon$-expansion is applied, not only to time, as is standard, but also to frequency. The paper presents the updated frequency–amplitude relationships and suggests that they appear more accurate, although it was not possible for this to be verified with numerical data. The motivation for expanding the frequency is solely to remove the secular terms in the response, and so the technique lacks the physical motivation that is present in the DNF method, as described in detail in the current paper.

Further attempts to detune the MS method have been proposed, though a number of these focus on the forced case in which it is common practice to perturb the forcing frequency [28, 29]. A more thorough investigation is given in [30], and a comparison of the MS method and the generalised method of averaging can be found in [31]. Additionally, the detuning applied in [32] has also been applied to the Lindstedt–Poincaré method of strained parameters and the generalised method of averaging, with these detuned methods producing identical truncated results [33].

In this paper, a comparison on the DNF and MS techniques is provided, with emphasis placed on the detuning used. Specifically, in Sect. 2, the two techniques are briefly outlined and compared using the Duffing oscillator as an example system, a system which is adopted in [27, 31–33]. The two techniques are equated by introducing a detuning step, which is physically interpreted as a perturbation about the response frequency rather than the linear frequency, into the MS technique in Sect. 3. The detuning approach employed in the DNF method will be applied in the MS method, and it will be shown that doing so allows the two methods to be equated. By considering a more general detuning, it is shown that using MS both the fundamental and the harmonic response predictions are affected by the detuning. This is in contrast to the DNF technique, in which only the harmonic response changes. In Sect. 4, the techniques are compared for a two-mode system, where it is shown that the techniques give the same results if the MS method is modified to include the detuning. Conclusions are drawn in Sect. 5.

## Approximate methods

This section introduces the DNF and MS techniques, giving an overview of how they are applied to a single-degree-of-freedom (SDOF) oscillator of the following form

1 $$\begin{aligned} \ddot{x}+\omega _n^2x+\varepsilon n_x(x)=0. \end{aligned}$$

Here, *x* denotes the displacement, $\omega _n$ represents the linear natural frequency, and $n_x(x)$ is a nonlinear term. For both techniques, the nonlinear term is assumed to be small. Here, this is indicated by $\varepsilon$, which may be thought of as a bookkeeping parameter that allows the relative size of terms to be tracked [8]. As such, $\varepsilon$ is taken to have a value of unity, such that it does not alter the equations. The application of the techniques is described as a series of steps, with the Duffing oscillator ($n_x(x)=\alpha x^3$) being used as an example.

### Direct normal form

The normal form approach is typically used to find periodic solutions to the equation of motion of a system. The objective of this approach is to apply a transform to the equation of motion to give a resonant form, in terms of transformed coordinate *u*, that can be solved exactly by using the following form for the solution, which assumes that system will respond as a single harmonic

2 $$\begin{aligned} u=u_p+u_m=\frac{A_c}{2}\mathrm {e}^{\mathrm {i}(\omega _r t-\phi _0)}+\frac{A_c}{2}\mathrm {e}^{-\mathrm {i}(\omega _r t-\phi _0)}. \end{aligned}$$

where $u_p$ and $u_m$ are used to denote the positive and negative parts of the exponents, respectively, and $A_c$ and $\omega _r$ represent the initial amplitude and nonlinear natural frequency, respectively. Time is denoted by *t* and $\phi _0$ denotes the phase of the response. Once *u* has been found, the harmonics of the response can be recovered using the transform equation.

The DNF approach is applied to equations of motion that are expressed in the linear modal coordinates, *q*, where $q=x$ for SDOF systems. This means that *x* could be used instead of *q* in the following equations. However, *q* has been kept to allow easier comparison with the MDOF case discussed in Sect. 4. The transform may be summarised as

3 $$\begin{aligned} \begin{aligned} \ddot{q}+\omega _n^2&q+\varepsilon n_q(q) =0 \xrightarrow {\displaystyle {q=u+\varepsilon \mathbf {h}_1\mathbf {u}^*_1+\varepsilon ^2\mathbf {h}_2\mathbf {u}^*_2} }\\&\ddot{u}+(\omega _r^2+\varepsilon \delta ) u+\varepsilon \mathbf {n}_{u1}\mathbf {u}^*_1+\varepsilon ^2\mathbf {n}_{u2}\mathbf {u}^*_2=0. \end{aligned}\nonumber \\ \end{aligned}$$

Here, $n_q(q)$ represents the small nonlinear terms of the untransformed equation and, as $q=x$ for a one degree-of-freedom system, $n_q(q)=n_x(q)$. In addition, the detuning $\omega _n^2=\omega _r^2 + \varepsilon \delta$, which will later be utilised in the MS method, is applied. The harmonics are now captured by the product of $\mathbf {h}_1$, a $1\times \ell$ vector of coefficients, and $\mathbf {u}^*_1$, an $\ell \times 1$ vector consisting of all the combinations of $u_p$ and $u_m$ that arise in $n_q(u_p+u_m)$; these harmonics are also assumed to be small. The method of finding the harmonics, $\mathbf {h}_1\mathbf {u}^*_1$, and the transformed nonlinear terms $\mathbf {n}_{u1}\mathbf {u}^*_1$ require three steps. These are now discussed, along with their application to the Duffing oscillator. An explanation of how both the steps and the detuning are derived is given in Appendix A, together with an indication of how they may be modified for MDOF systems.

By eliminating *q* from the original differential equation in Eq. (3) using the transform and then simplifying using the transformed equation of motion, the $\varepsilon ^i$ balance equation is given by the homological equation:

4 $$\begin{aligned} \varepsilon ^i:\quad -\mathbf {h}_i\ddot{\mathbf {u}}_i^*-\omega _r^2\mathbf {h}_i\mathbf {u}_i^*=\mathbf {n}_{ei}\mathbf {u}_i^* -\mathbf {n}_{ui}\mathbf {u}_i^*. \end{aligned}$$

The *excitation* of these equations, which defines the vectors $\mathbf {u}_i^*$, is given as

5 $$\begin{aligned}&\varepsilon ^1:\quad \mathbf {n}_{e1}\mathbf {u}_1^*=n_q(u),\end{aligned}$$

6 $$\begin{aligned}&\varepsilon ^2:\quad \mathbf {n}_{e2}\mathbf {u}_2^*=\delta \mathbf {h}_1\mathbf {u}_1^* +D\{n_q(u)\}\mathbf {h}_1\mathbf {u}_1^*, \end{aligned}$$

where $D\{n_q(u)\}$ represents the Jacobian of $n_q(u)$ and arises from the Taylor expansion of $\mathbf {n}(q)=\mathbf {n}(u+\varepsilon \mathbf {h}_1\mathbf {u}^*_1+\varepsilon ^2\mathbf {h}_2\mathbf {u}^*_2)$. These equations are solved using the following steps (which can be followed in Online Resource 1), first for the $\varepsilon ^1$ equation, as illustrated below, and then for the $\varepsilon ^2$ terms by making the necessary modification to the first step.

*Step *1$_{NF}$ The substitution $q = u = u_p + u_m$ is made in the nonlinear term to give $n_q(q) = n_q(u_p + u_m) = \mathbf {n}_{e1}\mathbf {u}^*_1$. Here, $\mathbf {n}_{e1}$ contains coefficient values and $\mathbf {u}^*_1$ is defined above.

For the Duffing oscillator, $n_q(u_p+u_m)=\alpha (u_p+u_m)^3$, giving

7 $$\begin{aligned} n_q(u_p + u_m)=\mathbf {n}_{e1}\mathbf {u}_1^*=\left[ \begin{array}{cccc} \alpha&\quad 3\alpha&\quad 3\alpha&\quad \alpha \end{array}\right] \left[ \! \begin{array}{c} u_p^3 \\ u_p^2u_m \\ u_pu_m^2\\ u_m^3\end{array}\!\right] .\nonumber \\ \end{aligned}$$

*Step *2$_{NF}$ Using Eq. (2), the variables $u_p$ and $u_m$ in $\mathbf {u}_1^*$ are written as a series of complex exponentials in time. The resulting vector is double differentiated with respect to time. The second derivative with respect to time can be expressed as a Hadamard product ($\circ$); $\mathrm {d}^2 \mathbf {u}_1^*/\mathrm {d} t^2 = -\mathbf {dd} \circ \mathbf {u}_1^*$. Further details on this are given in Appendix A.

For the Duffing example, using Eqs. (2) and (7), $\mathbf {u}_1^*$ may be written as

8 $$\begin{aligned} \mathbf {u}^*_1= \left[ \begin{array}{c} u_p^3\\ u_p^2u_m\\ u_pu_m^2\\ u_m^3\\ \end{array}\right] =\frac{A_c^3}{8} \left[ \begin{array}{c} \mathrm {e}^{\mathrm {i}3(\omega _{r}t-\phi _0)}\\ \mathrm {e}^{\mathrm {i}(\omega _{r}t-\phi _0)}\\ \mathrm {e}^{-\mathrm {i}(\omega _{r}t-\phi _0)}\\ \mathrm {e}^{-\mathrm {i}3(\omega _{r}t-\phi _0)}\\ \end{array}\right] , \end{aligned}$$

and so

9 $$\begin{aligned} \frac{\mathrm {d}^2\, \mathbf {u}_1^*}{\mathrm {d}\, t^2} = -\mathbf {dd} \circ \mathbf {u}_1^*, \,\text{ where } \quad \mathbf {dd}=\omega _r^2\left[ \begin{array}{cccc} 9&1&1&9\end{array}\right] ^\intercal .\nonumber \\ \end{aligned}$$

*Step *3$_{NF}$ Now, $\mathbf {h}_1$ and $\mathbf {n}_{u1}$ may be found using

10 $$\begin{aligned} (\mathbf {dd}^\intercal -\omega _r^2\mathbf {1}_{1,\ell })\circ \mathbf {h}_1=\mathbf {n}_{e1}-\mathbf {n}_{u1}, \end{aligned}$$

where $\mathbf {1}_{1,\ell }$ is a $1\times \ell$ row vector with every element being one. This expression is derived in Eq. (A.7) in Appendix A. For each nonzero element in the bracketed term, the corresponding value in $\mathbf {n}_{u1}$ is set to zero and the value in $\mathbf {h}_1$ is selected to satisfy the equation. For the zero elements in the bracketed term, the corresponding terms in $\mathbf {n}_{u1}$ are set to match those in $\mathbf {n}_{e1}$ and the $\mathbf {h}_1$ terms are set to zero. The result of this is a series of coefficients representing resonant terms in $\mathbf {n}_{u1}$ and harmonic (i.e. non-resonant) terms in $\mathbf {h}_1$.

For the Duffing oscillator, Eq. (10) becomes

11 $$\begin{aligned} \left( \omega _r^2\left[ \begin{array}{cccc}8&\quad 0&\quad 0&\quad 8\end{array}\right] \right) \circ \mathbf {h}_1\!=\!\left[ \begin{array}{cccc}\alpha&\quad 3\alpha&\quad 3\alpha&\quad \alpha \end{array} \right] -\mathbf {n}_{u1}. \nonumber \\ \end{aligned}$$

This allows us to find the required vectors

12 $$\begin{aligned} \mathbf {h}_1=\left[ \begin{array}{cccc}\displaystyle {\frac{\alpha }{8\omega _r^2}}&\quad 0&\quad 0&\quad \displaystyle {\frac{\alpha }{8\omega _r^2}}\end{array}\right] ,\quad \mathbf {n}_{u1}= \left[ \begin{array}{cccc}0&\quad 3\alpha&\quad 3\alpha&\quad 0\end{array}\right] . \nonumber \\ \end{aligned}$$

Note that the zeros on the left-hand side of Eq. (11) correspond to the resonant terms in Eq. (7) being set to zero, a feature that will also be observed in the MS method. Furthermore, it is important to note that there is some freedom of choice between the $\mathbf {h}_1$ and $\mathbf {n}_{u1}$ coefficients in Eq. (11). However, one of the advantages of this method is that the non-resonant terms, and only the non-resonant terms, in $\mathbf {u}^*$ are removed from the transformed equation of motion.

The near-identity transform to order $\varepsilon ^1$ may now be written as

13 $$\begin{aligned} \begin{aligned} q&=u+\varepsilon \mathbf {h}_1 \mathbf {u}^*_1\\&=\frac{A_c}{2}(\mathrm {e}^{\mathrm {i}(\omega _{r}t-\phi _0)} +\mathrm {e}^{-\mathrm {i}(\omega _{r}t-\phi _0)})\\&\quad +\varepsilon \left[ \frac{ \alpha }{8\omega _r^2}\;\; 0\;\; 0\;\; \frac{ \alpha }{8\omega _r^2} \right] \mathbf {u}_1^*\\&=A_c\cos (\omega _rt-\phi _0)\\&\quad +\varepsilon \frac{\alpha }{32 \omega _r^2} A_c^3 \cos (3(\omega _rt-\phi _0)). \end{aligned} \end{aligned}$$

From Eq. (3), along with Eq. (12), the transformed equation of motion may be written as

14 $$\begin{aligned} \ddot{u}+\omega _n^2 u+ \varepsilon 3\alpha \left( u_p^2u_m+u_pu_m^2\right) =0. \end{aligned}$$

To get the frequency–amplitude relationship for the backbone curve, we substitute the base solutions for $u_p$ and $u_m$ into Eq. (14) and then exactly balance either the $\mathrm {e}^{\mathrm {i}(\omega _r t-\phi _0)}$ or $\mathrm {e}^{-\mathrm {i}(\omega _r t-\phi _0)}$ terms (there are no non-resonant terms as these have been removed) to give

15 $$\begin{aligned} \omega _r= \sqrt{\omega _n^2 + \varepsilon \frac{3\alpha }{4}A_c^2}. \end{aligned}$$

This solution can be refined by repeating these steps, addressing the terms with increasing powers of $\varepsilon$ in turn. While each repetition leads to a more refined solution, they becoming increasingly onerous to perform algebraically. Thus, it is desirable to approach the true solution in the smallest possible number of iterations. This basis will be used to compare the DNF and MS methods in later in the paper.

To illustrate this refinement, if the $\varepsilon ^2$ terms are included in the near-identity transform by repeating the steps a second time, the following, more precise, solution can be obtained:

16 $$\begin{aligned} q= & {} A_c\cos (\omega _rt-\phi _0)\nonumber \\&+\,\varepsilon \frac{ \alpha }{32\omega _r^2}A_c^3\left( 1+\varepsilon \frac{3\alpha }{32\omega _r^2} A_c^2\right) \cos (3(\omega _rt-\phi _0))\nonumber \\&+\,\varepsilon ^2\frac{\alpha ^2}{512\omega _r^4}A_c^5 \cos (5(\omega _rt-\phi _0)). \end{aligned}$$

As a result, the frequency–amplitude relationship will now be given by

17 $$\begin{aligned} \omega _r^2= \omega _n^2 + \varepsilon \frac{3\alpha }{4}A_c^2+\varepsilon ^2 \frac{3\alpha ^2}{128\omega _r^2}A_c^4. \end{aligned}$$

### Multiple scales

The method of multiple scales is an established technique that is discussed at length in the literature (for example, see [22, 23, 26, 31, 32] and references therein), and here we provide a brief summary of this technique to form a basis on which modifications can be discussed later.

Following this review of the method, in Sect. 2.3, solutions found using the frequency detuning proposed in [32] will be presented; a more thorough investigation is given in Sect. 3, in which a comparison will be made between this detuning and that used in the DNF method.

The approach builds on the standard perturbation method in which the response is split into a series of terms with reducing significance $x=X_0+\varepsilon X_1 + \varepsilon ^2 X_2 + \cdots$. In MS, each of these time-dependent components are treated as functions of multiple timescales.

If these timescales are used, this response is assumed to be of the form

18 $$\begin{aligned} \begin{aligned} x(t)&=X_{0}(\tau , T, T_s)+\varepsilon X_{1}(\tau , T, T_s)\\&\quad +\varepsilon ^2 X_{2}(\tau , T, T_s)+\cdots \end{aligned} \end{aligned}$$

Here, the prescribed timescales are fast time over which oscillations occur, $\tau =\omega t$, a slower time over which the amplitudes evolve, $T=\varepsilon t$, and a timescale which is slower still, given by $T_s=\varepsilon ^2 t$. This definition of $\tau$, which incorporates frequency, is more typically associated with the Lindstedt–Poincaré method, but is applied here to allow a simpler comparison with the DNF method. These times, $\tau$, *T*, and $T_s$, are treated as independent variables, such that derivatives with respect to *t* can be expressed

19 $$\begin{aligned} \begin{aligned}&\frac{{\mathrm{d}}x}{\mathrm{d}t} =\omega \frac{\partial x}{\partial \tau }+\varepsilon \frac{\partial x}{\partial T} +\varepsilon ^2 \frac{\partial x}{\partial T_s},\quad \quad \\&\frac{{\mathrm{d}}^{2}x}{\mathrm{d}t^{2}}=\omega ^{2}\frac{\partial ^{2}x}{\partial \tau ^{2}}+ 2\omega \varepsilon \frac{\partial ^{2}x}{\partial T\partial \tau }+\varepsilon ^2\Big (\frac{\partial ^{2}x}{\partial T^2}+2\omega \frac{\partial ^{2}x}{\partial T_s\partial \tau }\Big ). \end{aligned}\nonumber \\ \end{aligned}$$

Note that fast time–frequency, $\omega$ is typically set to the linear natural frequency $\omega _n$, such that $\tau =\omega t=\omega _{n}t$. It is this selection of fast time that is now considered, and which gives the result listed in Table 1.

Substituting Eq. (19) into a general representation of an undamped, unforced nonlinear oscillator and using $\omega =\omega _{n}$, gives

20 $$\begin{aligned} \begin{aligned}&\ddot{x}+\omega _n^2 x+\varepsilon n_x(x) =0 \xrightarrow {\displaystyle {x=x(\tau ,\ T)} }\\&\quad \omega _{n}^{2}x^{\dag \dag }+ \varepsilon 2\omega _{n}x^{\dag \ddag }+\varepsilon ^2(x^{\ddag \ddag } + 2\omega _{n}x^{\dag *})\\&\quad +\omega _{n}^{2}x+\varepsilon n_x(x)=0, \end{aligned} \end{aligned}$$

where $\bullet ^\dag =\frac{\partial \bullet }{\partial \tau }$, $\bullet ^\ddag =\frac{\partial \bullet }{\partial T}$, and $\bullet ^* =\frac{\partial \bullet }{\partial T_s}$. Now, substituting Eq. (18) into the right-hand equation in Eq. (20), removing the terms of order $\varepsilon ^3$ and higher, and balancing for $\varepsilon$ lead to

21 $$\begin{aligned} \begin{array}{cl} \varepsilon ^0:&{} \omega _{n}^2 X_{0}^{\dag \dag }+\omega _{n}^2 X_{0}=0,\\ \varepsilon ^1:&{} \omega _{n}^2 X_{1}^{\dag \dag }+\omega _{n}^2 X_{1}= -2\omega _nX_{0}^{\dag \ddag } -n_x(X_0),\\ \varepsilon ^2:&{} \omega _{n}^2 X_{2}^{\dag \dag }+\omega _{n}^2 X_{2}=-2\omega _nX_{1}^{\dag \ddag } -X_{0}^{\ddag \ddag } -2\omega _nX_{0}^{\dag *}\\ &{}-D\{n_x(X_0)\}X_1. \end{array}\nonumber \\ \end{aligned}$$

To find the solution for the components of *x*, firstly the $\varepsilon ^0$ order balance in Eq. (21) is solved to give

22 $$\begin{aligned} X_{0}=A(T,T_s)\cos (\tau +\phi (T,T_s)), \end{aligned}$$

where $A(T,T_s)$ and $\phi (T,T_s)$ are slow time-varying amplitude and phase functions, respectively, which are defined by the initial conditions of the system. This allows the $\varepsilon ^1$ equation of Eq. (21) to be written as

23 $$\begin{aligned} \begin{aligned}&\omega _n^2 X_{1}^{\dag \dag }+ \omega _n^2 X_{1}=2\omega _n A(T,T_s)^{\ddag }\sin (\tau +\phi (T,T_s))\\&\quad + 2\omega _n A(T,T_s) \phi (T,T_s)^{\ddag }\cos (\tau +\phi (T,T_s))\\&\quad -n_x(X_0). \end{aligned} \end{aligned}$$

From Eq. (23), $A(T,T_s)$, $\phi (T,T_s)$, and $X_1$ may be calculated using the following steps (as demonstrated in Online Resource 2), written at $\varepsilon ^1$ order. These steps may then be reapplied to the $\varepsilon ^2$ balance in Eq. (21), to find the $\varepsilon ^2$ solution.

*Step *1$_{MS}$ The resonant terms, i.e. those that respond at $\tau =\omega _n t$ in Eq. (23), are removed and equated, writing

$$\begin{aligned} \begin{aligned}&2\omega _n A(T,T_s)^{\ddag }\sin (\tau +\phi (T,T_s))\\&\quad + 2\omega _n A(T,T_s) \phi (T,T_s)^{\ddag }\cos (\tau +\phi (T,T_s))\\&\quad ={\mathrm{Res}}\{n_{x}(X_0)\}, \end{aligned} \end{aligned}$$

*where* ${\mathrm{Res}}\{n_{x}(X_0)\}$ *represents the resonant terms in* $n_{x}(X_0)$. *This equation is then solved to find* $A(T,T_s)$ *and* $\phi (T,T_s)$.

For the Duffing oscillator example, we can write

24 $$\begin{aligned} \begin{aligned}&2\omega _n A(T,T_s)^{\ddag }\sin (\tau +\phi (T,T_s))\\&\qquad + 2\omega _n A(T,T_s) \phi (T,T_s)^{\ddag }\cos (\tau +\phi (T,T_s)) \\&\quad = {\mathrm{Res}}\left\{ \alpha A(T,T_s)^3\cos ^3(\tau +\phi (T,T_s))\right\} \\&\quad = \frac{3\alpha }{4}A(T,T_s)^3\cos (\tau +\phi (T,T_s)). \end{aligned} \end{aligned}$$

Balancing the $\sin (\tau +\phi )$ and $\cos (\tau +\phi )$ terms gives

25 $$\begin{aligned} \begin{aligned}&A(T,T_s)^{\ddag }=0,\quad \text{ and }\quad \\&\quad 2\omega _{n}A(T,T_s)\phi (T,T_s)^{\ddag } = \frac{3 \alpha }{4}A(T,T_s)^3, \end{aligned} \end{aligned}$$

respectively. These can be solved to give

26 $$\begin{aligned} \begin{aligned} A(T,T_s)&=A_c(T_s),\\ \phi (T,T_s)&=\frac{3\alpha }{8\omega _n}A_c(T_s)^2 T + \phi _c(T_s), \end{aligned} \end{aligned}$$

where $\phi _c$ is an integration constant representing a phase offset at $t=0$. Hence, using Eq. (22), we can write

27 $$\begin{aligned}&X_{0}=A_c(T_s)\cos \left( \omega _r t+\phi _c(T_s)\right) ,\,\,\nonumber \\&\quad \text{ with: }\,\, \omega _r=\omega _n+\varepsilon \frac{3\alpha }{8\omega _n}A_c(T_s)^2, \end{aligned}$$

where we have recalled that $\tau =\omega _n t$ and $T=\varepsilon t$ such that $\tau +\varepsilon \frac{3\alpha }{8\omega _n}A_c(T_s)^2 T=\omega _r t$.

*Step *2$_{MS}$ The remaining terms in Eq. (23),

$$\begin{aligned} \omega _n^2 X_{1}^{\dag \dag }+ \omega _n^2 X_{1}= -{\mathrm{NRes}}\{n_{x}(X_0)\}, \end{aligned}$$

*where* ${\mathrm{NRes}}\{n_{x}(X_0)\}$ *represents the non-resonant terms in* $n_{x}(X_0)$ *are now considered. Here the right-hand side may be viewed as an “excitation” of a linear dynamic system in* $X_1$ *which can be solved to generate harmonic responses terms in* *x*.

For the Duffing oscillator example, we have

28 $$\begin{aligned} \begin{aligned} \omega _n^2 X_{1}^{\dag \dag }+ \omega _n^2 X_{1}&=-{\mathrm{NRes}}\{n_{x}(X_0)\}\\&=-\frac{\alpha }{4}A_c(T_s)^3\cos \left( 3(\omega _r t\!+\!\phi _c(T_s))\right) . \end{aligned}\nonumber \\ \end{aligned}$$

where Eq. (27) has been used. Solving this linear differential equation gives

29 $$\begin{aligned} X_{1}=\frac{\alpha }{32\omega _n^2}A_c(T_s)^3\cos \left( 3(\omega _r t+\phi _c(T_s))\right) . \end{aligned}$$

Hence, the order $\varepsilon ^1$ solution, $x=X_0+\varepsilon X_1$, is given by

30 $$\begin{aligned} \begin{aligned} x&=A_c(T_s)\cos (\omega _r t+\phi _c)\\&\quad + \frac{ \alpha }{32\omega _n^2}A_c^3\cos (3(\omega _r t+\phi _c))\\&\text{ with: }\,\, \omega _r=\omega _n+\varepsilon \frac{3\alpha }{8\omega _n}A_c^2. \end{aligned} \end{aligned}$$

Here, we have written $A_c(T_s)=A_c$ and $\phi _c(T_s)=\phi _c$ as the timescale $T_s$ is not used to find the $\varepsilon ^1$ frequency–amplitude relationship.

As with the DNF methods, these steps can be repeated for higher-order $\varepsilon$ terms. Applying these to the $\varepsilon ^2$ terms, the refined solution is given by

31 $$\begin{aligned} \begin{aligned} x&=A_c\cos (\omega _r t+\phi _c) \\&\quad + \varepsilon \frac{ \alpha }{32\omega _n^2}A_c^3\Big (1 - \varepsilon \frac{21\alpha }{32\omega _n^2}A_c^2 \Big )\cos (3(\omega _r t+\phi _c))\\&\quad + \varepsilon ^2\frac{\alpha ^2 A_c^5}{1024\omega _n^4}\cos (5(\omega _r t+\phi _c)) \quad \\&\text{ with: }\quad \omega _r = \omega _n+\varepsilon \frac{3\alpha }{8\omega _n}A_c^2 - \varepsilon ^2\frac{15\alpha ^2}{256\omega _n^3}A_c^4. \end{aligned} \end{aligned}$$

Again, $A_c$ and $\phi _c$ are now a constants, though these would be functions of higher-order timescales if a higher $\varepsilon$-order solution was being sought.

### Duffing oscillator backbone curves

**Table 1:** Summary of approximate solutions and expressions for backbone curves for the undamped, unforced Duffing oscillator

Technique	Amplitude of fundamental	Amplitude of third harmonic	Amplitude of fifth harmonic
Direct normal form	$A_c$	$\displaystyle {\varepsilon \frac{ \alpha }{32\omega _r^2}A_c^3\left( 1+\varepsilon \frac{3\alpha }{32\omega _r^2}A_c^2\right) }$	$\displaystyle {\varepsilon ^2\frac{\alpha ^2}{512\omega _r^4}A_c^5}$
Multiple scales	$A_c$	$\displaystyle {\varepsilon \frac{ \alpha }{32\omega _n^2}A_c^3\Big (1 - \varepsilon \frac{21\alpha }{32\omega _n^2}A_c^2 \Big )}$	$\displaystyle {\varepsilon ^2\frac{\alpha ^2 }{1024\omega _n^4}A_c^5}$

**Fig. 1:**
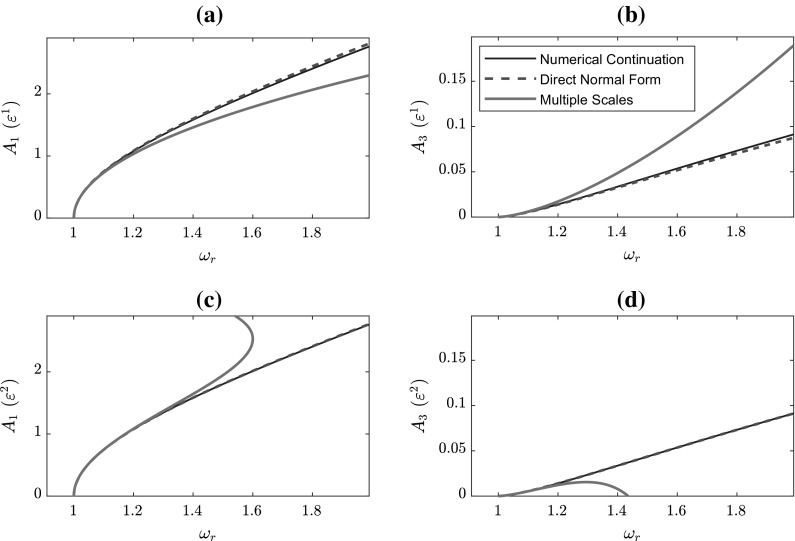
Comparison of first-order accurate ($\varepsilon ^1$) response curves found using approximate methods and numerical continuation for the undamped Duffing oscillator in terms of **a** the fundamental amplitude, **b** the third harmonic and **c** other harmonics, using $\omega _n=1$ and $\alpha =0.5$

It is now possible to compare the expressions for the frequency–amplitude relationship derived using two repetitions of the steps in each method. These are given by

32 $$\begin{aligned} \begin{array}{ll} \text{ DNF }: &{}\displaystyle {\omega _r^2= \omega _n^2 + \varepsilon \frac{3\alpha }{4}A_c^2+ \varepsilon ^2\frac{3\alpha ^2}{128\omega _r^2}A_c^4}, \\ \text{ MS }: &{}\displaystyle {\omega _r = \omega _n+\varepsilon \frac{3\alpha }{8\omega _n}A_c^2 - \varepsilon ^2\frac{15\alpha ^2}{256\omega _n^3}A_c^4}. \end{array} \end{aligned}$$

At this point, it is apparent that the DNF method detunes around the square of the response frequency, whereas the MS method directly detunes $\omega _r$. The corresponding higher harmonic response amplitudes are given in Table 1. Figure 1 presents the fundamental and third harmonic backbone curves for the Duffing oscillator at $\varepsilon ^1$- and $\varepsilon ^2$-order, along with the results found using the numerical continuation software Auto 07p [36]. In this figure, the influence that the type of detuning has on the results can be clearly seen. Both at $\varepsilon ^1$- and at $\varepsilon ^2$-order the DNF curve remains close to that of the numerical solution, whereas the backbone derived from the MS method diverges from this at higher amplitudes. Considering panels (a) and (c), it is evident that the $\varepsilon ^2$-order solution remains close to the numerical curve for a greater range of amplitudes, though this introduces a hardening-to-softening behaviour at higher values of $A_1$. Further, the harmonic components are poorly captured by the MS method in both panels (b) and (d).

The results displayed in Fig. 1 demonstrate the differences that can occur when a detuning is applied to the square of $\omega _r$, as opposed to directly to the linear term, and provide motivation for the application of the DNF detuning in the MS method, as described in Sect. 3.2. In particular, in contrast to the explicit form for the MS relationship, the DNF method gives an implicit equation in $\omega _r^2$. This can be easily rearranged to give a quadratic equation in $\omega _r^2$ which is easily solved and square rooted to give an explicit equation for $\omega _r$. This process becomes more complicated at higher orders of $\varepsilon$, at which point it is possible that either a Taylor expansion or numerical continuation would need to be used. That being said, the accuracy of the curves in Fig. 1 suggests that it is unlikely that these higher orders would be necessary to obtain a strong approximation of the true solution.

## Equating the techniques

In this section, we compare the derivations of the DNF and MS approaches. To do this, we first consider frequency detuning. The importance of this step for the DNF method was assessed in [27], in which the Duffing oscillator was used to demonstrate that it is this detuning which increases the accuracy of the technique in comparison with the classical normal form method. In light of the fact that perturbation methods repeat a specific set of steps to find a solution, as demonstrated in Sect. 2, we consider whether the same approach may be used in the MS method to improve the agreement with the DNF method at the same number of repetitions.

It should be noted that it is possible to introduce the intrinsic time-dependent amplitudes of the MS method to the DNF technique to allow transient behaviour to be captured. This is not investigated further here, as this paper focuses on the unforced, undamped behaviour of systems.

### Detuning the MS method

In the derivation of the DNF technique, a frequency detuning is applied, in which the square of the natural frequency is assumed to be detuned from the square of the response frequency such that the substitution $\omega _n^2=\omega _r^2+\varepsilon \delta$ can be made, where $\delta$ is introduced as a detuning parameter. This is discussed in Appendix A where, for multiple degrees of freedom, the equation is written ${\varvec{\Lambda }}={\varvec{\Gamma }}+\varepsilon {\varvec{\varDelta }}$. This allows the linear natural frequency to be replaced with the response frequency, $\omega _r$, and a detuning term, $\delta$, in the $\varepsilon ^1$ relationship, Eq. (A.3), and results in coefficients in ($\mathbf {dd}^\intercal -\omega _r^2\mathbf {1}_{1,\ell }$) expression that are exactly zero, see $Step 3_{NF}$.

This detuning has been discussed in [37], where it was shown that the detuning does not affect the frequency–amplitude relationship, but does improve the prediction of the third harmonic. The physical interpretation of this is associated with how the underlying linear system is defined—normally we consider the Duffing oscillator to have a linear stiffness term $\omega _n^2 x$ (and hence a natural frequency of $\omega _n$), but the same result can be achieved by treating the linear stiffness term as $\omega _r^2 x$ and modifying the nonlinear term to compensate for this, giving $\alpha x^3+\delta x$, where $\delta$ is a detuning parameter. Adopting the second approach can result in a smaller nonlinear term which more closely meets the key assumption that the non-linearity is of order $\varepsilon ^1$. Note that this interpretation of the detuning does not specifically rely on the assumption that $\delta$ is small, provided the new nonlinear term, $\alpha x^3 + \delta x$, remains small.

### Detuned multiple scales

Now let us consider how frequency detuning, which we will view as a means to express the equation of motion in terms of $\omega _r$, may be used in a MS approach, resulting in the *detuned multiple scales approach* (dMS). Firstly, when selecting the timescales we set the fast time as $\omega =\omega _r$ and hence $\tau =\omega _r t$. The result of this is that Eq. (20) is modified to

33 $$\begin{aligned} \begin{aligned}&\ddot{x}+\omega _n^2 x+\varepsilon n_x(x) =0 \xrightarrow {\displaystyle {x=x(\tau ,\ T)} }\\&\quad \omega _{r}^{2}x^{\dag \dag }+ \varepsilon 2\omega _{r}x^{\dag \ddag }+\varepsilon ^2x^{\ddag \ddag } +\omega _{n}^{2}x+\varepsilon n_x(x)=0, \end{aligned}\nonumber \\ \end{aligned}$$

where, now, $\tau =\omega _r t$, whereas previously, in Eq. (21), $\tau =\omega _n t$.

Now, we apply a frequency tuning to remove the $\omega _n$ terms. This tuning can take a number of forms, but let us select the same detuning as used in the DNF approach, with its link to modifying the linear and nonlinear stiffness terms, and use $\omega _n^2=\omega _r^2+\varepsilon \delta$. Substituting this and Eq. (18) into Eq. (33) and balancing for $\varepsilon ^i$ give

34 $$\begin{aligned} \begin{array}{cl} \varepsilon ^0:&{} \omega _{r}^2 X_{0}^{\dag \dag }+\omega _{r}^2 X_{0}=0, \\ \varepsilon ^1:&{} \omega _{r}^2 X_{1}^{\dag \dag }+\omega _{r}^2 X_{1}=-\delta X_0- 2\omega _rX_{0}^{\dag \ddag }- n_x(X_0), \end{array}\nonumber \\ \end{aligned}$$

which may be compared with Eq. (21) for the standard MS approach.

The solution to the $\varepsilon ^0$ equation is the same as before, namely Eq. (22), although note that now $\tau =\omega _r t$, whereas, previously, $\tau =\omega _n t$. The $\varepsilon ^1$ equation may be solved using the steps outlined previously. $Step 1_{MS}$ involves balancing the resonant term using the modified equation

35 $$\begin{aligned} \begin{aligned}&-\delta A(T)\cos (\tau +\phi (T))\\&\quad +\,2\omega _r A(T)^{\ddag } \sin (\tau +\phi (T))\\&\quad +\, 2\omega _r A(T) \phi (T)^{\ddag }\cos (\tau +\phi (T))={\mathrm{Res}}\{n_{x}(X_0)\}, \end{aligned}\nonumber \\ \end{aligned}$$

and for the Duffing oscillator this results in

36 $$\begin{aligned} \begin{aligned}&A(T)^\ddag =0,-\delta A(T)+2\omega _r A(T)\phi (T)^\ddag \\&\quad - \frac{3\alpha }{4}A(T)^3=0. \end{aligned} \end{aligned}$$

These can be solved to give

37 $$\begin{aligned} A(T)=\text{ constant }=A_c, \,\, \phi (T)=\phi _c, \,\, \delta =-\frac{3\alpha }{4}A_c^2.\nonumber \\ \end{aligned}$$

Note here, that the frequency shift is captured using $\delta$, as $\delta$ is defined as the detuning parameter, and so $\phi (T)$ is set to a constant. Using this and recalling that $\omega _n^2=\omega _r^2+\varepsilon \delta$ result in a frequency response equation given by

38 $$\begin{aligned} \omega _r = \sqrt{\omega _n^2+\varepsilon \frac{3\alpha }{4}A_c^2}. \end{aligned}$$

This is identical to the expression obtained by the DNF, as shown in Eq. (15) and Table 1.

Now, *Step *2$_{MS}$ is applied to find the harmonics captured by $X_1$. With the resonant terms removed, the $\varepsilon ^1$ balance may be expressed as

39 $$\begin{aligned} \begin{aligned} \omega _r^2 X_{1}^{\dag \dag }+ \omega _r^2 X_{1}&=-{\mathrm{NRes}}\{n_{x}(X_0)\}\\&=-\frac{\alpha }{4}A_c^3\cos \left( 3(\omega _r t+\phi _c)\right) , \end{aligned} \end{aligned}$$

where $X_0=A_c\cos (\tau +\phi _c)$ and $\tau =\omega _r t$ has been used. Solving this differential equation gives

40 $$\begin{aligned} X_{1}=\frac{\alpha }{32\omega _r^2}A_c^3\cos \left( 3(\omega _r t+\phi _c)\right) . \end{aligned}$$

Hence the order $\varepsilon ^1$ solution, $x=X_0+\varepsilon X_1$, is given by

41 $$\begin{aligned} x= & {} A_c\cos (\omega _r t+\phi _c) + \frac{ \alpha }{32\omega _r^2} A_c^3\cos (3(\omega _r t+\phi _c))\nonumber \\&\text{ with: }\quad \omega _r^2=\omega _n^2+\frac{3\alpha }{4}A_c^2. \end{aligned}$$

This is identical to the response predicted using the DNF approach, see Table 1.

As previously mentioned, a similar detuning of the MS technique is considered in [32], which introduces an $\varepsilon$ perturbation, $\omega ^2 = \omega _0^2+\varepsilon \omega _1+\varepsilon ^2\omega _2+\cdots$, to resolve the issues of secular terms in the response.^2^

Once truncated to order $\varepsilon ^1$, this expansion can be seen to be the same as that in the DNF method, though without the physical interpretation of a series expansion about the underlying natural frequency. It should be noted that, in [32], the first term is given as a square simply because it is convenient.

Note that the steps for the dMS method are illustrated in Online Resource 3.

### Comparison of detuned multiple scales and direct normal form

It has been shown that the predicted response using the DNF method can be matched by the dMS method. Now we compare these two techniques in more detail for the case where the amplitude of response is assumed to be fixed, i.e. $A(T)=A_c$. As with all the discussions up to this point, we will consider the $\varepsilon ^1$ accuracy case for a SDOF system.

The form of the response for the methods may be written as

42 $$\begin{aligned} \begin{aligned}&\text{ dMS: }\quad x=X_0+\varepsilon X_1\\&\text{ DNF: }\quad x=q=u+\varepsilon \mathbf {h}_1\mathbf {u}_1^*, \end{aligned} \end{aligned}$$

where the $\varepsilon ^0$ term on the right-hand side of both equations represents the resonant response and the $\varepsilon ^1$ term the harmonic response. The resonant response takes the form

$$\begin{aligned} \begin{aligned}&\text{ dMS: }\;\; X_0=A_c\cos (\tau +\phi _c), \;\; \tau =\omega _r t \\&\text{ DNF: }\;\; u=\frac{A_c}{2}\mathrm {e}^{\mathrm {i}(\omega _r t-\phi _c)}+\frac{A_c}{2}\mathrm {e}^{-\mathrm {i}(\omega _r t-\phi _c)} \\&\qquad \qquad \ =A_c\cos (\omega _r t+\phi _c). \end{aligned} \end{aligned}$$

Here we have set $\phi (T)=\phi _c$ as discussed in the previous section. In both cases, the expression for the response frequency is derived by considering the resonant terms in the $\varepsilon ^1$ equation.

For MS, for the case where $A(T)=A_c$ and $\phi (T)=\phi _c$, Eq. (35) can be simplified to give

43 $$\begin{aligned} -\delta A_c\cos (\omega _r t+\phi _c) = {\mathrm{Res}}\{n_{x}(A_c\cos (\omega _r t+\phi _c))\}, \nonumber \\ \end{aligned}$$

and hence, applying this in the dMS method and using $\omega _n^2=\omega _r^2+\varepsilon \delta$, we get

44 $$\begin{aligned} \begin{aligned} \omega _r^2&=\omega _n^2+\frac{1}{A_c\cos (\omega _r t+\phi _c)}\\&\quad {\mathrm{Res}}\{n_{x}(A_c\cos (\omega _r t+\phi _c))\}. \end{aligned} \end{aligned}$$

In the case of the DNF approach, the transformed dynamic equation is $\ddot{u}+\omega _n^2 u+\varepsilon \mathbf {n}_{u1}\mathbf {u}^*_1=0$ where $\mathbf {n}_{u1}\mathbf {u}^*_1$ is determined using $Step 3_{NF}$. This step solves $(\mathbf {dd}^\intercal -\omega _r^2\mathbf {1}_{1,\ell })\circ \mathbf {h}_1=\mathbf {n}_{q1}-\mathbf {n}_{u1}$ by considering the elements term by term. For elements where $(\mathbf {dd}^\intercal -\omega _r^2\mathbf {1}_{1,\ell })=0$, the resonant terms, the equation is satisfied by setting the corresponding elements in $\mathbf {n}_{u1}$ equal to those in $\mathbf {n}_{q1}$. This is equivalent to stating that $\mathbf {n}_{u1}\mathbf {u}^*_1=\text{ Res }\{\mathbf {n}_{q}\mathbf {u}^*_1\}=\text{ Res }\{n_q(q)\}$. By substituting this, along with the solution for *u* into the transformed equation of motion and noting that, for a SDOF system, $n_q=n_x$, we can write

45 $$\begin{aligned} \begin{aligned}&\left( -\omega _r^2+\omega _n^2\right) A_c\cos (\omega _r t+\phi _c)\\&\quad +\text{ Res }\{n_{x}(A_c\cos (\omega _r t+\phi _c))\}=0, \end{aligned} \end{aligned}$$

to obtain to the same expression as dMS, Eq. (43).

Now, considering the harmonic contribution, from Eq. (39), we have

46 $$\begin{aligned} \omega _r^2 X_{1}^{\dag \dag }+ \omega _r^2 X_{1}=-\text{ NRes }\{n_{x}(X_0)\}. \end{aligned}$$

Recalling for the dMS technique that fast time is defined as $\tau =\omega _r t$, the double derivative of $X_1$ with respect to $\tau$ may be written as $X_{1}^{\dag \dag }=(1/\omega _r^2)\ddot{X}_1$, hence

47 $$\begin{aligned} \text{ dMS: }\quad \frac{\mathrm {d}^2}{\mathrm {d}t^2}\{{X}_{1}\}+ \omega _r^2 X_{1}=-\text{ NRes }\{n_{x}(X_0)\}. \end{aligned}$$

In the case of the DNF method, the harmonic terms are found in *Step 3*$_{NF}$ where $(\mathbf {dd}^\intercal -\omega _r^2\mathbf {1}_{1,\ell })\circ \mathbf {h}_1=\mathbf {n}_{e1}-\mathbf {n}_{u1}$ is considered. For the non-resonant, or harmonic, elements this equation is satisfied by setting the left-hand side of the equation equal to the values in $\mathbf {n}_{e1}$ on an element-by-element basis. From the derivation in Appendix A, it can be seen that this solution arises from Eq. (A.3) and may be expressed as

48 $$\begin{aligned} -\mathbf {h}_1\ddot{\mathbf {u}}_1^*-\varGamma \mathbf {h}_1\mathbf {u}_1^*=\text{ NRes } \{\mathbf {n}_{e1}\mathbf {u}_1^*\}=\text{ NRes }\{n_x(u)\}.\nonumber \\ \end{aligned}$$

Recalling that, for a SDOF system, $\varGamma =\omega _r^2$ and that $\mathbf {h}_1$ is a coefficient matrix, we can rewrite Eq. (48) as

49 $$\begin{aligned} \text{ DNF: }\quad \frac{\mathrm {d}^2}{\mathrm {d}t^2}\{\mathbf {h}_1\mathbf {u}_1^*\}+\omega _r^2\mathbf {h}_1\mathbf {u}_1^*=-\text{ NRes }\{n_x(u)\}. \end{aligned}$$

It can be seen that this is the same as the harmonic expression for the direct MS approach, Eq. (46), by following the relationship in Eq. (42) and setting $u = X_0$ and $\mathbf {h}_1\mathbf {u}_1^* = X_1$.

From this, we can conclude that, at an accuracy level of $\varepsilon ^1$, the prediction of periodic oscillations using the DNF and MS methods can be made the same. This requires the MS technique to use $\tau =\omega _r t$, as in the DNF method, for fast time and to remove $\omega _n$ from the equations of motion using the frequency tuning $\omega _n^2=\omega _r^2+\varepsilon \delta$. As discussed in [37], this is justified based on the idea that the system can be linearised about a stiffness $\omega _r^2 x$ rather than $\omega _n^2 x$ to potentially reduce the size of the nonlinear term. This may be substituted into Eq. (41) to give the full solution to order $\varepsilon ^1$.

### Alternative frequency tunings

So far in this section, we have shown that the MS and DNF techniques are equivalent, to order $\varepsilon ^1$, under the special conditions that the fast time is set to $\tau =\omega _r t$ and the stiffness term, $\omega _n^2 x$, in the equation of motion is rewritten as $(\omega _r^2 + \varepsilon \delta )x$, where $\delta$ can still be viewed as a detuning parameter. However, this frequency tuning approach raises the question about the predicted response when a different detuning parameter is selected.

For the case of the DNF method, this has been addressed in [37] for both single- and multi-degree-of-freedom systems. Consider the arbitrary frequency tuning $\omega _n^2=\omega _d^2+\delta _d$, where $\omega _d$ is the detuned frequency with $\omega _d=\omega _r$ for the standard technique described in 2.1. In the MDOF notation used in Appendix A, the equivalent expression is ${\varvec{\Lambda }}={\varvec{\Gamma }}_d+{\varvec{\varDelta }}_d$. In [37], it was shown that the prediction of the response at the fundamental frequency is independent of the chosen detuning at order $\varepsilon ^1$. The reason for this is that the only change to the $\varepsilon ^1$ balance, Eq. (A.3), is that ${\varvec{\Gamma }}_d$, a diagonal matrix of $\omega _{ri}^2$ terms, is replaced by a diagonal matrix of $\omega _{di}^2$ terms. The result is that, in $Step 3_{NF}$, $\mathbf {h}_1$ and $\mathbf {n}_{u1}$ are now found using Eq. (10).

When satisfying Eq. (10) using the arbitrary frequency tuning, we apply the rule defined in $Step 3_{NF}$ to entries that are approximately zero in the brackets, rather than looking for values which are exactly zero. The corresponding terms in $\mathbf {n}_{u1}$ are still set to those in $\mathbf {n}_{q1}$. As these terms are the same as those for the case where $\omega _d=\omega _r$, the resulting nonlinear function in *u*, $\mathbf {n}_{u1}$, also remains the same. Hence, the $\varepsilon ^1$ order equation of motion in *u*, and the subsequent response at the resonant frequency, is independent of the selection of $\omega _d$. However, the harmonic response prediction is affected, as each term in this is dependent on the non-near-zero value of the bracketed term in Eq. (10). The result is that, for the Duffing oscillator, the vector for $\mathbf {h}_1$ becomes

50 $$\begin{aligned} \mathbf {h}_1=\left[ \begin{array}{cccc}\displaystyle {\frac{\alpha }{9\omega _r^2-\omega _d^2}}&\quad 0&\quad 0&\quad \displaystyle {\frac{\alpha }{9\omega _r^2-\omega _d^2}}\end{array}\right] \end{aligned}$$

which may be compared to Eq. (12) for the case where the standard frequency tuning, $\omega _d=\omega _r$, is used. Thus, in contrast to the fundamental frequency response, which is not a function of the detuning parameter, the prediction for the third harmonic amplitude is dependent on the choice of detuning frequency and is given by

51 $$\begin{aligned} A_3=\frac{\alpha A_c^3}{4(9\omega _r^2-\omega _d^2)}. \end{aligned}$$

**Fig. 2:**
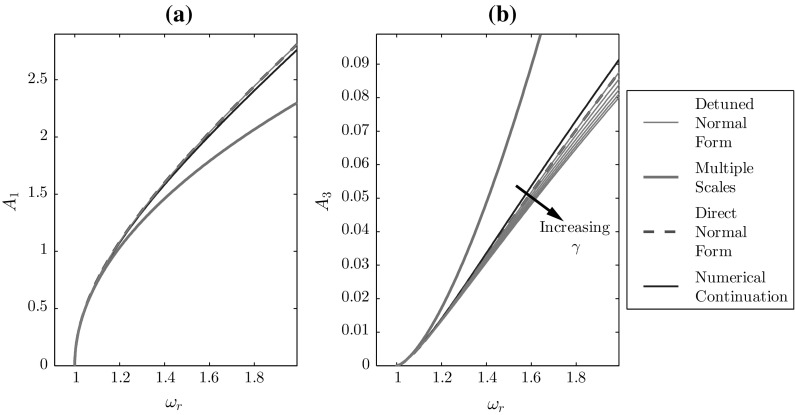
**a** Fundamental and **b** third harmonic amplitude response curves for the undamped Duffing oscillator, using $\omega _n=1$, $\alpha =0.5$, and $\gamma \in \left[ 0,1\right]$

Figure 2 shows the DNF prediction of the response of the Duffing oscillator in terms of the first and third harmonics for a range of frequency tuning frequencies, $\omega _d=\omega _r+(\omega _n-\omega _r)\gamma$, from $\gamma =0$, corresponding to the standard detuning used in DNF (i.e. $\omega _d=\omega _r$) to $\gamma =1$, where no detuning is used (i.e. $\omega _d=\omega _n$). Figure 2a shows that the prediction of the response at the resonant frequency is robust to the choice of detuning parameter; however, the third harmonic response is affected by its choice and is better captured using the standard DNF detuning ($\gamma =0$) than with no detuning ($\gamma =1$).

Now, considering MS, we have already seen that the selection of the fast time–frequency and the subsequent frequency tuning equation (for the case where this frequency did not match $\omega _n$) does affect both the resonant response and that of the harmonics at order $\varepsilon ^1$ accuracy. In general, if we write the fast time as $\tau =\omega _d t$, where for dMS $\omega _d=\omega _r$, the $\varepsilon$ balance equations [see Eq. (21) for MS and Eq. (34) for dMS] become

52 $$\begin{aligned} \begin{array}{cl} \varepsilon ^0:&{} \omega _{d}^2 X_{0}^{\dag \dag }+\omega _{d}^2 X_{0}=0, \\ \varepsilon ^1:&{} \omega _{d}^2 X_{1}^{\dag \dag }+\omega _{d}^2 X_{1}=\delta _d X_0- 2\omega _d X_{0}^{\dag \ddag }- n_x(X_0), \end{array}\nonumber \\ \end{aligned}$$

where the $\varepsilon ^i \omega _n^2 X_i$ terms have been rewritten as $\varepsilon ^i \omega _d^2 X_i+\varepsilon ^{i+1}\delta _d X_i$ prior to the balancing using the $\omega _n^2=\omega _d^2+\delta _d$ frequency tuning expression. In addition, the Taylor series expansion $\omega _n=\omega _d+\varepsilon \delta _d/(2\omega _d)$ has been used to remove $\omega _n$ in the slow dynamics term $2\omega _n X_{0}^{\dag \ddag }$.

Following this approach results in $X_0=A(T)\cos (\tau +\phi (t))=A(T)\cos (\omega _d t+\phi (t))$ and, using the $\varepsilon ^1$ equation, we find that, for the Duffing oscillator,

53 $$\begin{aligned} \begin{aligned}&A(T)^\ddag =0,-\delta _d A(T)+2\omega _d A(T)\phi (T)^\ddag \\&\quad - \frac{3\alpha }{4}A(T)^3=0, \end{aligned} \end{aligned}$$

which may be compared to Eqs. (25) and (36) for the MS and dMS techniques, respectively. Solving the differential equations in $\phi (T)$ and *A*(*T*) and substituting the solutions into the $X_0$ expression give

54 $$\begin{aligned} \begin{aligned} X_0&=A(T)\cos (\omega _d t+\phi (t))\\&=A_c\cos \left( \left[ \omega _d +\frac{\delta _d}{2\omega _d}+\frac{3\alpha }{8\omega _d}A_c^2\right] t+\phi _c\right) , \end{aligned} \end{aligned}$$

where $A_c$ and $\phi _c$ are the values of *A*(*T*) and $\phi (T)$ at $t=0$. Recalling that $\delta _d$ is defined in $\omega _n^2=\omega _d^2+\delta _d$, this gives the response frequency $\omega _r=\omega _n^2/(2\omega _d)+\omega _d/2+3\alpha A_c^2/(8\omega _d)$. Writing $\omega _d=\omega _r+(\omega _n-\omega _r)\gamma$ results in the response frequency equation

55 $$\begin{aligned} \begin{aligned}&(1-\gamma ^2)\omega _r^2+(2\omega _n\gamma ^2)\omega _r\\&\quad -\left( \omega _n^2(1+\gamma ^2)+\frac{3\alpha A_c}{4}\right) =0, \end{aligned} \end{aligned}$$

and, from solving the $\varepsilon ^1$ expression, the resulting harmonic response amplitude is

56 $$\begin{aligned} A_3=\frac{\alpha A_c^3}{32\omega _d^2}=\frac{\alpha A_c^3}{32(\omega _r+\gamma (\omega _n-\omega _r))^2} \end{aligned}$$

**Fig. 3:**
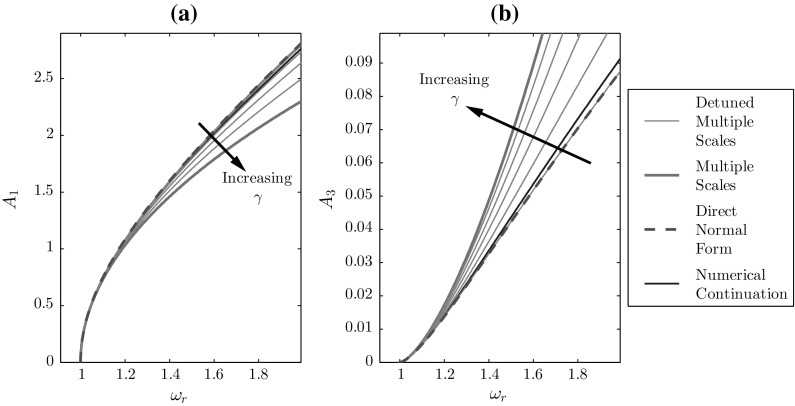
**a** Fundamental and **b** third harmonic amplitude response curves for the undamped Duffing oscillator, using $\omega _n=1$, $\alpha =0.5$, and $\gamma \in \left[ 0,1\right]$

Figure 3 demonstrates that varying $\gamma$ from 0 to 1 transforms the response from the DNF/dMS to the standard MS response. For the MS technique, $\delta _d=0$ and, hence, the frequency shift away form $\omega _n$ is captured by $\phi (T)$. However, for the dMS technique, $\omega _d=\omega _r$ and so $\phi (T)=\phi _c$ represents the fact that the $X_0$ response is at response frequency $\omega _r$. These represent two special cases, for a general frequency tuning with fast time $\tau =\omega _d t$; the thin, green curves in Fig. 3 represent a continuum between these two cases. Note that the accuracy of the DNF method is only reached when the detuning from that method is used. Interestingly, the fundamental response is independent of the detuning for the DNF method, whereas this is not the case for MS.

## Example: non-symmetric, two-mass oscillator

A 2DOF system is considered in this section, allowing the two methods to be compared using a more complex system, as well as examining the robustness of the frequency tuning methods.

The system under consideration is the same as that in [38]. It consists of a two-mass oscillator with a symmetric underlying system of linear springs; two cubic nonlinear springs are added in parallel with the corresponding linear springs, one grounding the first mass and one connecting the two masses. Therefore, the force-deflection equation for the grounding of the first mass is $F=k_1(\varDelta x)+\kappa _1(\varDelta x)^3$ and the relationship is similar for the springs connecting the masses, given by $F=k_2(\varDelta x)+\kappa _2(\varDelta x)^3$. Here, $k_i$ and $\kappa _i$ are the spring constants of the linear and nonlinear springs, respectively. As in [38], both techniques are applied directly to the modal equations of motion to ease the comparison of solutions. These are given by

57 $$\begin{aligned} {\ddot{\mathbf{q}}}+{\varvec{\Lambda }}{} \mathbf q +\mathbf N _q(\mathbf q )=\mathbf 0 \end{aligned}$$

where ${\varvec{\Lambda }}$ is a diagonal matrix of the squared natural frequencies of the underlying linear system, $\omega _{ni}^2$, and

58 $$\begin{aligned} \mathbf N _q(\mathbf q )=\frac{\kappa _1 }{2m }\begin{pmatrix} (q_1+q_2)^3\\ (q_1+q_2)^3+\beta q_2^3 \end{pmatrix}, \end{aligned}$$

with $$\beta =16 \frac{\kappa _2 }{\kappa _1 }$$.

The application of the methods is largely the same as for the Duffing oscillator considered in previous sections, so only a brief overview is given below. For brevity, solutions will only be considered to order $\varepsilon ^1$. In addition, we provide scripts, as supplementary material, that allow the equations to be derived symbolically using Wolfram Mathematica.

### Multiple scales

Standard perturbations are again implemented, giving the two modal coordinates as

59 $$\begin{aligned} \begin{aligned}&q_1(t)=Q_{10}(\tau _1,\ T)+\varepsilon Q_{11}(\tau _1,\ T)+\cdots ,\\&q_2(t)=Q_{20}(\tau _2,\ T)+\varepsilon Q_{21}(\tau _2,\ T)+\cdots . \end{aligned} \end{aligned}$$

The notation $\tau _i = \omega _{ni} t$ has been introduced to ensure that the fast time for each mode corresponds to the appropriate natural frequency. It should be noted that, for this model, we consider the case $\tau _1 \approx \tau _2$.

Implementing this perturbation, as well as the corresponding adaptation of the derivative given in Eq. (19), results in zeroth- and first-order perturbation equations that take the same form as in Eq. (21), and hence, the former can be solved to give

60 $$\begin{aligned} \begin{aligned}&Q_{10}=A_{1,1}(T)\cos (\tau _1+\phi _1(T)),\\&Q_{20}=A_{2,1}(T)\cos (\tau _2+\phi _2(T)), \end{aligned} \end{aligned}$$

**Fig. 4:**
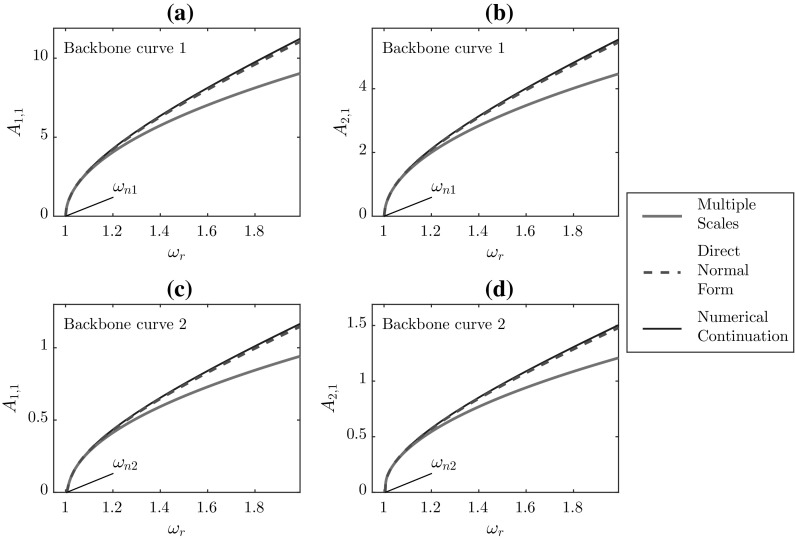
Fundamental amplitude response curves for the 2DOF system, using $\omega _{n1}=1$, $\omega _{n2}=1.005$, $\kappa _1=0.5$, and $\kappa _2=0.05$. Panels **a** and **c** show the response of the first mode, and panels **b** and **d** show the response of the second mode

where $$A_{i,j}$$ denotes the amplitude of the $$jth $$ harmonic in the $$ith $$ mode. These solutions can be applied to the first-order equation [equivalent to the SDOF equation in Eq. (21)] to give the $$\varepsilon ^1$$ equations

61 $$\begin{aligned}&\omega _{n1}^2 Q_{11}^{\dag \dag }+\omega _{n1}^2 Q_{11}\nonumber \\&\quad = 2\omega _{n1} A_{1,1}(T)^{\ddag }\sin (\tau _1+\phi _1(T))\nonumber \\&\qquad +\frac{A_{1,1}(T)}{8}(16\omega _{n1}\phi _1^{\ddag }(T)\nonumber \\&\qquad -3\kappa _1[A_{1,1}(T)^2+2A_{2,1}(T)^2])\cos (\tau _1+\phi _1(T))\nonumber \\&\qquad -n_{q1}(Q_{10},Q_{20}),\nonumber \\&\qquad \omega _{n2}^2 Q_{21}^{\dag \dag }+\omega _{n2}^2 Q_{21}\nonumber \\&\quad = 2\omega _{n2} A_{2,1}(T)^{\ddag }\sin (\tau _2+\phi _2(T))\nonumber \\&\qquad + \frac{A_{2,1}(T)}{8}(16\omega _{n1}\phi _2^{\ddag }(T) -3\kappa _1[A_{1,1}(T)^2\nonumber \\&\qquad +2(1+\beta )A_{2,1}(T)^2])\cos (\tau _2+\phi _2(T))\nonumber \\&\qquad -n_{q2}(Q_{10},Q_{20}). \end{aligned}$$

The nonlinear terms, $n_{qi}(Q_{10},Q_{20})$, are lengthy and contain fundamental and harmonic terms. Therefore, they are not shown here.

Collecting the resonant terms allows the amplitudes and phases to be calculated, as in the SDOF case, so that

62 $$\begin{aligned} \begin{aligned}&2\omega _{n1} A_{1,1}(T)^{\ddag }\sin (\tau _1+\phi _1(T))\\&\quad +\frac{A_{1,1}(T)}{8}(16\omega _{n1}\phi _1^{\ddag }(T)\\&\quad -3\kappa _1[A_{1,1}(T)^2+2A_{2,1}(T)^2])\cos (\tau _1+\phi _1(T))\\&\quad -Res \{n_{q1}(Q_{10},Q_{20})\}=0,\\&2\omega _{n2} A_{2,1}(T)^{\ddag }\sin (\tau _2+\phi _2(T))\\&\quad + \frac{A_{2,1}(T)}{8}(16\omega _{n1}\phi _2^{\ddag }(T) -3\kappa _1[A_{1,1}(T)^2\\&\quad +2(1+\beta )A_{2,1}(T)^2])\cos (\tau _2+\phi _2(T))\\&\quad -Res \{n_{q2}(Q_{10},Q_{20})\}=0. \end{aligned} \end{aligned}$$

These equations can now be solved to give:

63 $$\begin{aligned} \begin{aligned} A_{1,1}(T)&=A_{c1},\\ \quad \phi _1(T)&=\frac{3\kappa _1}{16\omega _{n1} A_{c1}}(A_{c1}+A_{c2})^3 T + \phi _{c1},\\ A_{2,1}(T)&=A_{c2},\\ \phi _2(T)&=\frac{3\kappa _1}{16\omega _{n2} A_{c2}}((A_{c1}\!+\!A_{c2})^3 + \beta A_{c2}^3) T\!+\! \phi _{c2}, \end{aligned}\nonumber \\ \end{aligned}$$

where $\beta = \frac{16\kappa _2}{\kappa _1}$. Note that the expressions for phase enforce the condition that neither fundamental amplitude can be equal to zero. Therefore, recalling that $\tau _1=\omega _{n1} t$ results in

64 $$\begin{aligned} \begin{aligned}&Q_{10}=A_{c1}\cos (\omega _r t + \phi _{c1}),\\&\,\, with: \quad \omega _r = \omega _{n1} + \varepsilon \frac{3\kappa _1}{16\omega _{n1} A_{c1}}(A_{c1}+A_{c2})^3,\\&Q_{20}=A_{c2}\cos (\omega _r t + \phi _{c2}),\\&\,\, with: \quad \omega _r = \omega _{n2}\\&\quad + \varepsilon \frac{3\kappa _1}{16\omega _{n2} A_{c2}}\left[ (A_{c1}+A_{c2})^3 + \beta A_{c2}^3\right] . \end{aligned} \end{aligned}$$

This leads to the following compatibility condition

65 $$\begin{aligned}&\omega _{n1} \omega _{n2}(\omega _{n2}-\omega _{n1})\nonumber \\&\quad =\frac{3\varepsilon \kappa _1}{16 A_{c1} A_{c2}}\left[ (A_{c2}\omega _{n2}-A_{c1}\omega _{n1})(A_{c1}+A_{c2})^3 \right. \nonumber \\&\qquad \left. -\beta A_{c1} A_{c2}^3\omega _{n1}\right] . \end{aligned}$$

This expression can now be used to find $A_{c1}$ in terms of $A_{c2}$, or vice versa. However, the explicit solution is non-trivial and is not shown here.

The resulting backbone curves from Eq. (64) are given in Fig. 4 and discussed in Sect. 4.3. Due to the involved process required to find the harmonics, analytical solutions for these are not given, but have been derived using Wolfram Mathematica and solved numerically to allow comparison between the techniques; this is discussed in Sect. 4.3.

### Direct normal form

This technique also closely mirrors its SDOF counterpart, so only a brief description of the key differences is given. The resonant equations of motion are once again found in terms of $\mathbf u$, with

66 $$\begin{aligned} u_i=u_{ip}+u_{im}=\frac{A_{ci}}{2}(\mathrm {e}^{+\mathrm {i}(\omega _r t-\phi _i)}+\mathrm {e}^{-\mathrm {i}(\omega _r t-\phi _i)}).\nonumber \\ \end{aligned}$$

**Fig. 5:**
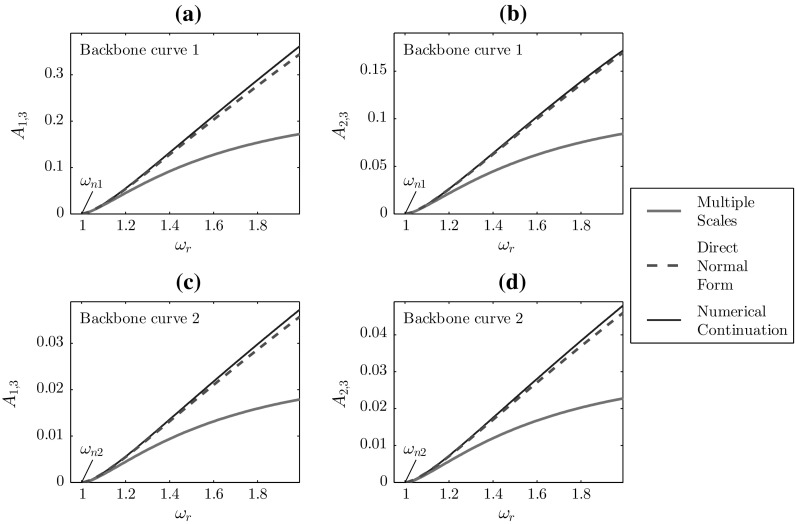
Third harmonic amplitude response curves for the 2DOF system, using $\omega _{n1}=1$, $\omega _{n2}=1.005$, $\kappa _1=0.5$, and $\kappa _2=0.05$. Panels **a** and **c** show the response of the first mode, and panels **b** and **d** show the response of the second mode

$Steps 1_{NF}$–$3_{NF}$ are followed as previously described and are not shown here due to the large size of the matrices involved. Full workings are shown in [38].

The frequency–amplitude relationships that arise are given by

67 $$\begin{aligned} \begin{aligned}&\frac{3 \kappa _1 (A_{c1}+A_{c2}){}^3}{8 m}+A_{c1} (\omega _{n1}^2 -\omega _r ^2)=0,\\&\frac{3\kappa _1 [(A_{c1}+A_{c2}){}^3+\beta A_{c2}^3]}{8 m}+A_{c2} (\omega _{n2}^2-\omega _r ^2)=0. \end{aligned}\nonumber \\ \end{aligned}$$

These results are comparable with Eq. (64) and the resulting backbone curves are, again, displayed in Fig. 4. Similarly, the equations for the harmonics are algebraically complex and are solved numerically.

### Detuned multiple scales

The key difference when applying the dMS in two degrees of freedom (2DOF) is that separate frequency tunings are to be applied to each mode

68 $$\begin{aligned} \omega _{ni}^2=\omega _r^2+\varepsilon \delta _i, \quad \text {for~} i=1,2. \end{aligned}$$

Again, the resonant equations are used to find the amplitude, phase, and now detuning parameter. For the 2DOF case under consideration, these are given by

69 $$\begin{aligned} \begin{aligned}&A_{1,1}(T)=A_{c1}, \quad \phi _1(T)=\phi _{c1},\\&\quad \delta _1=\frac{3\kappa _1}{8\omega _{n1} A_{c1}}(A_{c1}+A_{c2})^3,\\&A_{2,1}(T)=A_{c2}, \quad \phi _2(T)=\phi _{c2},\\&\quad \delta _2 = \frac{3\kappa _1}{8\omega _{n2} A_{c2}}\left[ (A_{c1}+A_{c2})^3 + \beta A_{c2}^3\right] . \end{aligned} \end{aligned}$$

Thus, substituting these values into Eq. (68) gives the frequency–amplitude equations as

70 $$\begin{aligned} \begin{aligned}&\frac{3 \kappa _1 (A_{c1}+A_{c2}){}^3}{8 m}+A_{c1} \left( \omega _{n1}^2-\omega _r ^2\right) =0,\\&\frac{3 \kappa _1 \left( (A_{c1}+A_{c2}){}^3+\beta A_{c2}^3\right) }{8 m}+A_{c2} \left( \omega _{n2}^2-\omega _r ^2\right) =0. \end{aligned}\nonumber \\ \end{aligned}$$

Comparing these with Eq. (67) demonstrates that the results from the dMS method, once again, match those from DNF. It should be made clear that, in Figs. 4 and 5, the curve for the dMS method has not been printed as it is coincident with the DNF curve.

As with the SDOF case, the final forms of $q_i$ are identical, though this is not shown here for reasons of brevity.

### Comparison of the techniques

The fundamental backbone curves for the first and second modal responses are given in Fig. 4. Four backbone curves are shown for each technique. Panels (a) and (b) correspond to the first backbone curve of the system, that is, the curve which initiates at the first natural frequency of the underlying linear system, $\omega _{n1}$; panels (c) and (d) represent the second backbone curve. These results are comparable to those for the Duffing oscillator in Fig. 1, with the MS curve underestimating the numerical continuation results and the DNF/dMS results again remain closer to the numerical continuation results. The difference between the methods grows significantly with increasing amplitude. In particular, the MS results diverge noticeably from the numerical and DNF/dMS counterparts at higher amplitudes. As verified in [38], this is the result of the loss of influence of the higher-order terms during the linearisation of the system.

Interestingly, the third harmonic components of the backbone curves in Fig. 5 are qualitatively different from the equivalent curve for the Duffing oscillator. While the amplitudes of the third harmonics from the MS method in the SDOF case were greater than those from numerical continuation, Fig. 5 shows that the opposite is true for the 2DOF responses. This inconsistency suggests that the MS method is less robust to changes in the system compared to the DNF and dMS methods, which remains consistent across the two cases, although higher-order cases have not been considered in this study.

## Conclusions and discussion

This paper presents a comparison between the multiple scales and direct normal form techniques and investigates whether the two methods can produce equivalent results. In particular, the detuning used in the DNF method was applied in the MS method to investigate whether a similar level of accuracy could be achieved. The frequency detuning, which can be physically interpreted as a way of reducing the amplitude of the nonlinear term based on adapting the effective linear stiffness, is inherent in the DNF method and has been shown to improve the prediction of the harmonic response content. In applying this detuning in the MS method, it was shown that the two methods could be equated, giving identical solutions up to $\varepsilon ^2$ order.

The DNF is advantageous insofar as a natural detuning approach is intrinsic in its formulation, whereas this is not the case for the MS technique. It is, therefore, the decision of the user as to whether a detuning is utilised to increase the accuracy of the method. Furthermore, it has been demonstrated that the fundamental response prediction is robust to changes in detuning in the DNF method. Since this is not the case for the MS technique, we observe that there is room for further optimisation of the detuning to be applied, which could further increase the accuracy of the method.

To aid the understanding of these methods, as well as the differences in their implementation, Wolfram Mathematica files for the 2DOF case have been provided as open access data files. These closely follow the steps defined in Sect. 2 and are designed to be used in conjunction with this paper to give a practical understanding of each procedure.

### Electronic supplementary material

Below is the link to the electronic supplementary material.

Supplementary material 1 (nb 111 KB)

Supplementary material 2 (nb 112 KB)

Supplementary material 3 (nb 41 KB)

## Appendix A

Considering a MDOF system, expressed in linear modal coordinates, the direct normal form, to order $\varepsilon$ accuracy, involves the transform

A.1 $$\begin{aligned} \begin{aligned} \ddot{\mathbf {q}}+\varLambda \mathbf {q}+\varepsilon \mathbf {n}_q(\mathbf {q}) =0&\xrightarrow {\displaystyle {\mathbf {q}=\mathbf {u}+\varepsilon \mathbf {h}_1\mathbf {u}^*_1} }\\&\ddot{\mathbf {u}}+\varLambda \mathbf {u}+\varepsilon \mathbf {n}_{u1}\mathbf {u}^*_1=0. \end{aligned} \end{aligned}$$

Here, $\varLambda$ is a diagonal matrix with the $i^\mathrm {th}$ diagonal element being the square of the $i^\mathrm {th}$ linear natural frequency, $\omega _{ni}^2$. This reduces to Eq. (3) for the case where a single mode is considered, with $\mathbf {q}=q$, $\mathbf {u}=u$, $\mathbf {n}=n$ and $\varLambda =\omega _{n1}^2=\omega _n^2$ to indicate that the terms are now scalar quantities or functions.

To find vector $\mathbf {u}^*_1$, we make use of the fact that in the transformed equation of motion the harmonic terms have been removed such that the response in the $i^\mathrm {th}$ coordinates $u_i$ may be expressed as

A.2 $$\begin{aligned} \begin{aligned} u_i&=u_{pi}+u_{mi}\\&=\frac{A_{ci}}{2}\mathrm {e}^{\mathrm {i}(\omega _{ri} t-\phi _{0i})}+\frac{A_{ci}}{2}\mathrm {e}^{-\mathrm {i}(\omega _{ri} t-\phi _{0i})}. \end{aligned} \end{aligned}$$

Using this the nonlinear function $\mathbf {n}_q$ expressed in terms of $\mathbf {u}$ may be written as $\mathbf {n}_q(\mathbf {u})=\mathbf {n}_q(\mathbf {u}_p+\mathbf {u}_m)=\mathbf {n}_{e1}\mathbf {u}_1^*$. Here $\mathbf {u}^*$ is an $\ell \times 1$ vector consisting of all $\ell$ combinations of $u_{pi}$ and $u_{mi}$ generated by $\mathbf {n}_q(\mathbf {u}_p+\mathbf {u}_m)$ and $\mathbf {n}_{e1}$ is a $n\times \ell$ matrix (for an *n* degree-of-freedom system) that contains coefficient values. The subscript *e* in $\mathbf {n}_{e1}$ indicates that this term can be thought of as an *excitation* term in the following discussion.

Now, considering Eq. (A.1), the transform can be substituted into the equation of motion in $\mathbf {q}$. The resulting expression can first be simplified using the resonant equation of motion and then balanced in terms of $\varepsilon$ to give

A.3 $$\begin{aligned} \varepsilon ^1:&s -\mathbf {h}_1\ddot{\mathbf {u}}_1^*-\varGamma \mathbf {h}_1\mathbf {u}_1^*=\mathbf {n}_{e1} \mathbf {u}_1^*-\mathbf {n}_{u1}\mathbf {u}_1^*, \end{aligned}$$

where $\varGamma$ is a diagonal matrix with the $i^\mathrm {th}$ diagonal element being the square of the $i^\mathrm {th}$ response frequency, $\omega _{ri}^2$. Here, a Taylor series expansion has been used on the nonlinear function: $\mathbf {n}(\mathbf {q})=\mathbf {n}(\mathbf {u})+\mathcal {O}(\varepsilon )$. In addition, a form of frequency tuning, similar to that used in [37], is employed: we write $\varLambda =\varGamma +\varepsilon \varDelta$ when considering the $\mathbf {h}_1\mathbf {u}_1^*$ terms.

Now, observing that each element in $\mathbf {u}^*_1$ is made up of $\mathbf {u}_p$ and $\mathbf {u}_m$ elements which themselves are complex exponentials in time, if follows that each term in $\mathbf {u}^*_1$ may be written as complex exponentials in time. Therefore, when differentiating $\mathbf {u}^*$ twice with time, each element maps onto a scaled version of itself. So, we may represent $\ddot{\mathbf {u}}^*_1$ as

A.4 $$\begin{aligned} \frac{\mathrm {d}^2\, \mathbf {u}_1^*}{\mathrm {d}\, t^2} = -\mathbf {dd} \circ \mathbf {u}_1^* \end{aligned}$$

where $\mathbf {dd}$ is a vector of length $\ell \times 1$ and $\circ$ is the Hadamard product (element-wise matrix multiplication). Using this, we can write the first term in Eq. (A.3) as

A.5 $$\begin{aligned} -\mathbf {h}_1\ddot{\mathbf {u}}^*_1=\mathbf {h}_1\left( \mathbf {dd}\circ \mathbf {u}^*_1\right) = \left[ \left( \mathbf {1}_{n,1}\mathbf {dd}^\intercal \right) \circ \mathbf {h}_1\right] \mathbf {u}^*_1,\nonumber \\ \end{aligned}$$

where $\mathbf {1}_{a,b}$ is an $a\times b$ matrix of ones. In addition, making use of the fact that $\varGamma$ is a diagonal matrix, the second term in Eq. (A.3) may be expressed in a similar form

A.6 $$\begin{aligned} \varGamma \mathbf {h}_1\mathbf {u}_1^*=\left[ \left( \varGamma \mathbf {1}_{n,\ell }\right) \circ \mathbf {h}_1\right] \mathbf {u}_1^*. \end{aligned}$$

Substituting these into Eq. (A.3) gives

A.7 $$\begin{aligned} (\mathbf {1}_{n,1}\mathbf {dd}^\intercal -\varGamma \mathbf {1}_{n,\ell })\circ \mathbf {h}_1=\mathbf {n}_{e1}-\mathbf {n}_{u1}. \end{aligned}$$

It can be seen that this reduces to the equation given in $Step 3_{NF}$ of the normal form description for the SDOF case ($n=1$). Using $Step 3_{NF}$, the equation can be solved to find $\mathbf {h}_1$ and $\mathbf {n}_{u1}$ and hence identify the transform and transformed equation of motion, respectively.

## Footnotes

1 In some previous papers, this method is called “second-order normal form”, which is a phrase that is open to more than one possible meaning, so we choose to avoid it here.

2 Interestingly, it can be seen that $\omega _0=\omega _n$, even though the $\omega _i$ terms are arbitrary in [32].
